# Adequacy of the Sequential-Task Paradigm in Evoking Ego-Depletion and How to Improve Detection of Ego-Depleting Phenomena

**DOI:** 10.3389/fpsyg.2016.00136

**Published:** 2016-02-09

**Authors:** Nick Lee, Nikos Chatzisarantis, Martin S. Hagger

**Affiliations:** Psychology and Behavioural Medicine Research Group, School of Psychology and Speech Pathology, Faculty of Health Sciences, Curtin UniversityPerth, WA, Australia

**Keywords:** self-regulation, self-control, strength model, methodology, sequential-task, dual-task, effort

Self-control is defined as individuals' capacity to alter, modify, change, or override impulses, desires, and habitual responses (Baumeister, 2002; Muraven et al., 2005). Capacity for self-control is important and adaptive. Without it, we would be “slaves” to habits and impulses and unable to engage in sustained, goal-directed behavior. Loss of self-control has been shown to be related to numerous maladaptive health, social, and economic outcomes (Baumeister, 2002). Contemporary theories indicate that human capacity for self-control is limited (Baumeister et al., 1998). According to the strength model of self-control, performance on tasks requiring self-control draws energy from a general, unitary, and limited “internal” resource (Baumeister et al., 1998; Muraven et al., 1998). Because this resource is finite, the model predicts that engaging in tasks requiring self-control would lead to the depletion of the resource and reduced performance on subsequent self-control tasks. The state of self-control resource depletion is termed “ego-depletion.”

Baumeister and colleagues tested the strength model of self-control using a “sequential-task” paradigm in which participants engaged in two consecutive tasks. For participants allocated to an experimental (ego-depletion) group, both tasks required self-control, while only the second task required self-control for participants in the control (no depletion) group (Baumeister et al., 2007). Reduced performance on the second task in participants assigned to the ego-depletion group compared to participants in the control group constituted support for the ego-depletion effect. Examples of self-control tasks typically used in sequential-task paradigm experiments include downregulating emotions while watching emotionally-arousing video-clips (e.g., Muraven et al., 1998) or suppressing an unwanted thought by attending to a distracter (e.g., Muraven et al., 2006). An initial meta-analysis revealed a medium effect size (*d* = 0.62) across 198 tests of the ego-depletion effect using the sequential-task paradigm (Hagger et al., 2010).

Recent analyses have suggested that Hagger et al.'s meta-analytic evidence for the ego-depletion effect might have been inflated due to publication bias. Re-analyses of previous findings (Carter and McCullough, 2013, 2014) and a new meta-analysis including unpublished studies (Carter et al., 2015) revealed substantial “small study” bias in the effect size reported in Hagger et al.'s (2010) original meta-analysis. There have also been studies that failed to replicate the ego-depletion effect (e.g., Xu et al., 2014), or found it to be substantially smaller in size than reported in meta-analytic syntheses (e.g., Tuk et al., 2015). Carter et al. (2015) concluded that given an unbiased review, the ego-depletion effect was not distinguishable from zero. Overall, this evidence raises questions as to the existence of the ego-depletion effect.

Despite this, Hagger and Chatzisarantis (2014) suggested that rather than rejecting the strength model outright, it may be more informative to test the specific conditions under which ego-depletion might occur through an analysis of potential moderators. A first step to identifying these moderators may be revisit the sequential-task experimental paradigm adopted in almost all ego-depletion studies. The paradigm has become the accepted method to test ego-depletion without being subjected to substantive critical examination. The sequential-task paradigm, while elegant, has inherent problems. A critical assumption of the paradigm is that participants exert sufficient effort to deplete their self-control resources on the first self-control task (Chatzisarantis and Hagger, 2015b) and there has been little attention paid to ascertaining participants' level of exertion during the first task beyond subjective assessments of effort and fatigue (Chatzisarantis and Hagger, 2015a). If participants do not engage in effortful self-control on the first task, it is unlikely that they will be sufficiently depleted to show decrements in performance on subsequent tasks, leading to null or greatly reduced ego-depletion effect.

Determination of effort invested in the first task is likely to be strongly influenced by an individual's motivation to consciously alter the habitual or impulsive response. Wegner and Zanakos (1994) suggested that people make the effort to overcome a habitual response and exert self-control only when the response is consciously accessible. Gaining control over responses that are typically automatic and occur beyond an individual's awareness determines whether an individual will be effective in taking appropriate action to overcome the response. For example, people who are instructed to supress a thought will only engage in thought suppression if they actively search for a distracter whenever the unwanted thought to be supressed emerges into consciousness. Self-control in this context is, therefore, a function of the number of times individuals make the effort to overcome a habitual response whenever a habitual response emerges into consciousness. It follows that if individuals are not sufficiently motivated to overcome an impulse or a counter-intentional response when performing a self-control task then cannot be considered to engage in an act of self-control (Wegner and Zanakos, 1994), and as a result ego-depletion will not occur.

The importance of motivation to self-control is shared in a number of recent alternative models of the ego-depletion effect which implicate motivation as a critical determinant (e.g., Inzlicht and Schmeichel, 2012; Kurzban et al., 2013; Loftus et al., 2015). For example, Inzlicht and Schmeichel (2012) propose that when individuals invest substantial effort in self-control acts, they experience an involuntary decrease in motivation to exert control and increased motivation to act through less effortful and more impulsive pathways, and a shift in attention away from the need to maintain control and attend more to cues that grant immediate gratification. These shifts undermine the person's ability to maintain self-control manifested in reduced task performance. Therefore, rather than depletion being the result of the lack of resources, it is the consequences of the shift in the individuals motivational and attentional focus. While the perspective of mechanism differs from the resource depletion account, motivation is central to Inzlicht and Schmeichel's model of ego-depletion effect. Failure to invest a high degree of effort in the initial task in the sequential-task paradigm may offset losses in motivation and attention shifts in subsequent tasks.

Considering the evidence and these perspectives on self-control, the exertion of sufficient effort on the first task of the sequential-task paradigm is, therefore, critical in determining whether ego-depletion is induced. Insufficient effort may mean that participants fail to exert self-control and expend resources. Although previous research has taken subjective measures of effort on the first task, few have attempted to formally manipulate motivation on the first task. This may be critical determinant as to whether participants exert sufficient effort on the first task and may add considerable methodological variance to the ego-depletion literature.

A tacit assumption of studies adopting the sequential-task paradigm is that participants are sufficiently motivated to engage in the first task. However, it is possible that participants are not motivated to invest sufficient effort in the first task to trigger ego-depletion. For various reasons, participants may vary in the extent to which they are motivated to engage in the tasks, which may introduce considerable variance to performance on the first task and affect subsequent task performance. This might be overcome by introducing a methodological element to the sequential-task paradigm that induces participants to invest identical effort levels on the first task. Providing participants with an incentive to engage in the first task such as providing a monetary reward would be expected to increase motivation and effort on the first task. Previous studies have only employed monetary incentives for the second task to alleviate the ego-depletion effect (e.g., Muraven and Slessareva, 2003). Such methods have yet to be applied to motivate participants in the first task. Introducing the incentive would be expected to lead to participants being equally motivated to engage in the task and expend sufficient self-control resources to evoke depletion.

While we are aware that the use of an extrinsic incentive such as a monetary reward may undermine motivation through the overjustification effect (Lepper et al., 1973; Hagger and Chatzisarantis, 2011), we do not expect this to apply to the sequential-task experiment design. Monetary rewards are motivating as long as the rewards are omnipresent and the undermining of motivation would only apply on contexts where the reward is removed and participants given free-choice to engage in the task. Furthermore, the impact that monetary reward have on the first task for the depletion group, would also have the same effect on the non-depletion group. The overjustification effect is therefore controlled for through the experimental design. Also experiments using this approach should measure intrinsic motivation at baseline and control for any variation in subsequent analyses.

The sequential-task paradigm might also have led to inconsistencies in detecting the ego-depletion effect because it leaves the duration of self-control exertion on the first task unspecified. Ego-depletion may not be observed in the second task if the duration of the first task is of sufficient duration to expend self-control resources. This is consistent with Hagger et al.'s (2010) meta-analysis which showed duration of the first task to have a weak moderating effect on the ego-depletion effect size. In addition, research on mental fatigue literature may also provide support for this position as studies inducing mental fatigue require participants to engage in demanding, effortful, or monotonous tasks for prolonged periods (Boksem and Tops, 2008). Similar to ego-depletion research, evidence indicates that mental fatigue could impede function in multiple domains of self-control including attention (Boksem et al., 2005) and cognition (Lorist, 2008). It is, however, worth noting is that although the mental fatigue literature may be informative of the ego-depletion effect, particularly on the effects of task duration on depletion, there is a fundamental difference in the methods adopted to investigate the effects. The sequential-task paradigm requires a control group that engages in a task that require no self-control. Studies in fatigue on the other hand often set the control group with the similar task but with less time or with reduced, but never absent, demands. Although the methods used in mental fatigue research may be informative of the effect of duration on self-control over time, they do not show whether duration exacerbates ego-depletion effects because they do not include a no-depletion control condition of equal duration.

The differences in methods used in the mental fatigue and ego-depletion literatures notwithstanding, duration on the initial task may be a determining factor of whether depletion is induced. We propose a series of studies adopting the sequential-task paradigm in which the duration of the initial task is manipulated systematically. This may shed light on whether variation in the depletion effect across studies is attributable to variations in first task duration. It would also be important to conduct such studies in tasks from multiple domains of self-control to demonstrate the domain generality and indicate whether duration interacts with task domain in inducing depletion. Such research may assist in developing guidelines for the minimum duration required for the first task of the sequential-task paradigm to reliably induce ego-depletion.

In conclusion, while recent evidence questions the ego-depletion effect, we contend that there are inherent limitations in the sequential-task paradigm which may affect whether or not ego-depletion is detected. Self-control failure occurs when individuals engage in tasks that require overcoming an impulse or counter-intentional response over a period of time. However, such failure is dependent on participants investing sufficient effort on self-control tasks to evoke depletion. Previous research adopting the sequential task paradigm makes the tacit assumption that individuals invest equal and sufficient effort on the initial task, but, to date, no research has formally evaluated this assumption. We contend that to reliably induce depletion, sequential-task paradigm experiments need to provide sufficient motivation for individuals invest effort in the first task and that the first task is of sufficient duration. We have proposed two modifications to the sequential task paradigm: provide incentives, and extend the duration of the first task. We call for a series of experiments in which researchers examine the effects of introducing incentives for the first task in the dual-task paradigm on ego-depletion, and a set of experiments in which duration of the first task is systematically varied. Such research will confirm our current proposals for standardized procedures in sequential task paradigm experiments that can reliably induce ego-depletion and may contribute to resolving the observed variation in the size of the effect.

## Author contributions

NL was the primary author of this article with suggestions and help from his doctoral supervisors, NC and MH.

### Conflict of interest statement

The authors declare that the research was conducted in the absence of any commercial or financial relationships that could be construed as a potential conflict of interest.

## References

[B1] Baumeister R. F. (2002). Ego depletion and self-control failure: an energy model of the self's executive function. Self Identity 1, 129–136. 10.1080/152988602317319302

[B2] Baumeister R. F. Bratslavsky E. Muraven M. Tice D. M. (1998). Ego depletion: is the active self a limited resource? J. Pers. Soc. Psychol. 74, 1252–1265. 10.1037/0022-3514.74.5.1252 9599441

[B3] Baumeister R. F. Vohs K. D. Tice D. M. (2007). The strength model of self-control. Curr. Dir. Psychol. Sci. 16, 351–355. 10.1111/j.1467-8721.2007.00534.x12079249

[B4] Boksem M. A. Meijman T. F. Lorist M. M. (2005). Effects of mental fatigue on attention: an ERP study. Cogn. Brain Res. 25, 107–116. 10.1016/j.cogbrainres.2005.04.011 15913965

[B5] Boksem M. A. Tops M. (2008). Mental fatigue: costs and benefits. Brain Res. Rev. 59, 125–139. 10.1016/j.brainresrev.2008.07.001 18652844

[B6] Carter E. C. Kofler L. M. Forster D. E. McCullough M. E. (2015). A series of meta-analytic tests of the depletion effect: self-control does not seem to rely on a limited resource. J. Exp. Psychol. 144, 796–815. 10.1037/xge0000083 26076043

[B7] Carter E. C. McCullough M. E. (2013). Is ego depletion too incredible? Evidence for the overestimation of the depletion effect. Behav. Brain Sci. 36, 683–684. 10.1017/S0140525X13000952 24304780

[B8] Carter E. C. McCullough M. E. (2014). Publication bias and the limited strength model of self-control: has the evidence for ego depletion been overestimated? Front. Psychol. 5:823. 10.3389/fpsyg.2014.00823 25126083 PMC4115664

[B9] Chatzisarantis N. L. Hagger M. S. (2015a). Illusionary delusions. Willingness to exercise self-control can mask effects of glucose on self-control performance in experimental paradigms that use identical self-control tasks. Appetite 84, 322–324. 10.1016/j.appet.2014.10.025 25450893

[B10] Chatzisarantis N. L. Hagger M. S. (2015b). Unsuccessful attempts to replicate effects of self-control operations and glucose on ego-depletion pose an interesting research question that demands explanation. Appetite 84, 328–329. 10.1016/j.appet.2014.10.024 25450895

[B11] Hagger M. S. Chatzisarantis N. L. D. (2011). Causality orientations moderate the undermining effect of rewards on intrinsic motivation. J. Exp. Soc. Psychol. 47, 485–489. 10.1016/j.jesp.2010.10.010

[B12] Hagger M. S. Chatzisarantis N. L. D. (2014). It is premature to regard the ego-depletion effect as “too incredible”. Front. Psychol. 5:298 10.3389/fpsyg.2014.00298 PMC3989757 24782802

[B13] Hagger M. S. Wood C. Stiff C. Chatzisarantis N. L. D. (2010). Ego depletion and the strength model of self-control: a meta-analysis. Psychol. Bull. 136, 495–525. 10.1037/a0019486 20565167

[B14] Inzlicht M. Schmeichel B. J. (2012). What is ego depletion? Toward a mechanistic revision of the resource model of self-control. Pers. Psychol. Sci. 7, 450–463. 10.1177/1745691612454134 26168503

[B15] Kurzban R. Duckworth A. L. Kable J. W. Myers J. (2013). An opportunity cost model of subjective effort and task performance. Behav. Brain Sci. 36, 661–679. 10.1017/S0140525X12003196 24304775 PMC3856320

[B16] Lepper M. R. Greene D. Nisbett R. E. (1973). Undermining children's intrinsic interest with extrinsic rewards: a test of the “overjustification” hypothesis. J. Pers. Soc. Psychol. 28, 129–137. 10.1037/h0035519

[B17] Loftus A. M. Yalcin O. Baughman F. D. Vanman E. J. Hagger M. S. (2015). The impact of transcranial direct current stimulation on inhibitory control in young adults. Brain Behav. 5:e00332. 10.1002/brb3.332 25874165 PMC4389055

[B18] Lorist M. M. (2008). Impact of top-down control during mental fatigue. Brain Res. 1232, 113–123. 10.1016/j.brainres.2008.07.053 18687317

[B19] Muraven M. Collins R. L. Shiffman S. Paty J. A. (2005). Daily fluctuations in self-control demands and alcohol intake. Psychol. Addict. Behav. 19, 140. 10.1037/0893-164X.19.2.140 16011384

[B20] Muraven M. Shmueli D. Burkley E. (2006). Conserving self-control strength. J. Pers. Soc. Psychol. 91, 524. 10.1037/0022-3514.91.3.524 16938035

[B21] Muraven M. Slessareva E. (2003). Mechanisms of self-control failure: motivation and limited resources. Pers. Soc. Psychol. Bull. 29, 894–906. 10.1177/0146167203029007008 15018677

[B22] Muraven M. Tice D. M. Baumeister R. F. (1998). Self-control as limited resource: regulatory depletion patterns. J. Pers. Soc. Psychol. 74, 774–789. 10.1037/0022-3514.74.3.774 9523419

[B23] Tuk M. A. Zhang K. Sweldens S. (2015). The propagation of self-control: self-control in one domain simultaneously improves self-control in other domains. J. Exp. Psychol. 144, 639–654. 10.1037/xge0000065 25822462

[B24] Wegner D. M. Zanakos S. (1994). Chronic thought suppression. J. Pers. 62, 615–640. 10.1111/j.1467-6494.1994.tb00311.x 7861307

[B25] Xu X. Demos K. E. Leahey T. M. Hart C. N. Trautvetter J. Coward P.. (2014). Failure to replicate depletion of self-control. PLoS ONE 9:e109950. 10.1371/journal.pone.0109950 25333564 PMC4204816

